# Supplementary material for: Large spatial extension of the zero-energy Yu–Shiba–Rusinov state in a magnetic field
Source: Nat Commun. 2020 Apr 14;11:1834. doi: 10.1038/s41467-020-15322-9 (PMC7156378; doi:10.1038/s41467-020-15322-9)
Supplement: Supplementary file 1 — Supplementary Information [file 41467_2020_15322_MOESM1_ESM.pdf]

SUPPLEMENTARY NOTE 1. CONDUCTANCE OF THE QUANTUM DOT

Supplementary Figure 1 shows the differential conductance of the quantum dot in an even larger gate voltage window than in the main text. White dashed rectangle marks the region presented in Fig. 3b of the main text. In the extended gate region the measurement indicates double dot behavior. The resonances corresponding to the second dot are almost vertical, i.e. the second dot is better coupled to g_B gate than to g_P, accordingly it is positioned further from the superconducting electrode. The signatures of the second dot are less pronounced in the conductance of the tunnel probe (not shown), in accordance with the larger distance from the superconductor. In the main text we focused on the marked region, where the stability diagram exhibits a single quantum dot behavior.

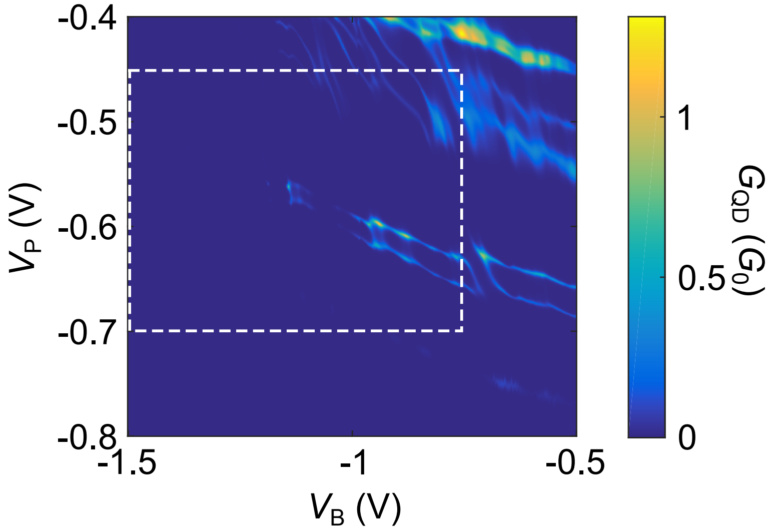

Supplementary Figure 1. Differential conductance of the quantum dot as the function of the barrier gate voltage V_B and plunger gate voltage V_P. The white dashed rectangle marks the region presented in Fig. 3b of the main text.

SUPPLEMENTARY NOTE 2. SHIBA STATE AT FINITE ENERGY

The characterization of the Shiba state was carried out at finite bias conditions too. Such measurement is presented on Supplementary Figure 2. Panel a (b) shows the conductance of the tunnel probe (quantum dot) as the function of the plunger gate voltage, V_P and the symmetrically applied bias voltage, V_{bias} (see Fig. 5 of the main text for the electric circuit). Panel b is identical to Fig. 3c of the main text with the usual eye-shaped signature of the Shiba state.

The differential conductance of the tunnel probe, G_T, strongly depends on the applied bias voltage, it increases significantly at finite bias up to 120 μV and fluctuates at larger bias. This is due to the fact that in our case the tunnel probe is not an ideal insulator barrier, but an InAs nanowire section. However, note that as long as the analysis is restricted to the low bias regime, where the conductance is suppressed, the nanowire can be treated as a tunnel electrode.

With the plunger gate the Shiba state can be tuned to finite energy, where one expects the conductance enhancement to appear at finite bias in G_T, similar to the case of STM measurements (see e.g. Supplementary Reference 1). Indeed one can find the signature of the Shiba state at finite bias voltages, indicated by the black arrows on panel a. However, among other features it is less pronounced and its magnitude varies with the plunger gate voltage. Therefore, we decided to focus on the zero-energy Shiba state in the main text.

---

[1] A. Yazdani, B. A. Jones, C. P. Lutz, M. F. Crommie, and D. M. Eigler, Science **275**, 1767 (1997), ISSN 0036-8075, https://science.sciencemag.org/content/275/5307/1767.full.pdf, URL `https://science.sciencemag.org/content/275/5307/1767`.

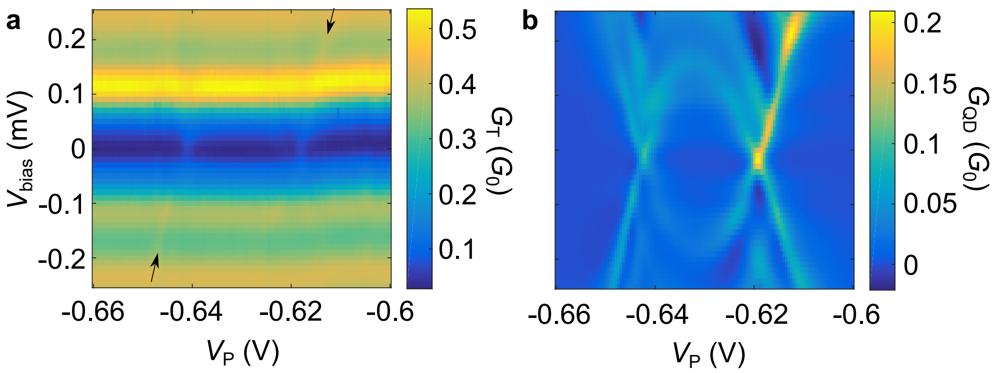

Supplementary Figure 2. Simultaneous, finite-bias measurement of the tunnel probe (panel a) and the quantum dot (panel b). The right panel is the same as Fig. 3c of the main text. The Shiba-related conductance enhancement is also present in G_{T} at finite bias, but it is less pronounced.
